# Characterizing Antibody Responses to *Plasmodium vivax* and *Plasmodium falciparum* Antigens in India Using Genome-Scale Protein Microarrays

**DOI:** 10.1371/journal.pntd.0005323

**Published:** 2017-01-24

**Authors:** Swapna Uplekar, Pavitra Nagesh Rao, Lalitha Ramanathapuram, Vikky Awasthi, Kalpana Verma, Patrick Sutton, Syed Zeeshan Ali, Ankita Patel, Sri Lakshmi Priya G., Sangamithra Ravishankaran, Nisha Desai, Nikunj Tandel, Sandhya Choubey, Punam Barla, Deena Kanagaraj, Alex Eapen, Khageswar Pradhan, Ranvir Singh, Aarti Jain, Philip L. Felgner, D. Huw Davies, Jane M. Carlton, Jyoti Das

**Affiliations:** 1 Center for Genomics and Systems Biology, Department of Biology, New York University, New York, NY, United States of America; 2 National Institute of Malaria Research, Indian Council of Medical Research, Sector 8, Dwarka, New Delhi, India; 3 National Institute of Malaria Research Field Unit, Sector 1 Health Center, Raurkela, Odisha, India; 4 National Institute of Malaria Research Field Unit, Civil Hospital, Nadiad, Gujarat, India; 5 National Institute of Malaria Research Field Unit, Indian Council of Medical Research, National Institute of Epidemiology Campus, Ayapakkam, Chennai, Tamil Nadu, India; 6 Department of Medicine, Division of Infectious Diseases, University of California Irvine, Irvine, CA, United States of America; University of Sao Paulo, BRAZIL

## Abstract

Understanding naturally acquired immune responses to *Plasmodium* in India is key to improving malaria surveillance and diagnostic tools. Here we describe serological profiling of immune responses at three sites in India by probing protein microarrays consisting of 515 *Plasmodium vivax* and 500 *Plasmodium falciparum* proteins with 353 plasma samples. A total of 236 malaria-positive (symptomatic and asymptomatic) plasma samples and 117 malaria-negative samples were collected at three field sites in Raurkela, Nadiad, and Chennai. Indian samples showed significant seroreactivity to 265 *P*. *vivax* and 373 *P*. *falciparum* antigens, but overall seroreactivity to *P*. *vivax* antigens was lower compared to *P*. *falciparum* antigens. We identified the most immunogenic antigens of both *Plasmodium* species that were recognized at all three sites in India, as well as *P*. *falciparum* antigens that were associated with asymptomatic malaria. This is the first genome-scale analysis of serological responses to the two major species of malaria parasite in India. The range of immune responses characterized in different endemic settings argues for targeted surveillance approaches tailored to the diverse epidemiology of malaria across the world.

## Author Summary

Although malaria deaths have fallen by 60% worldwide since 2000, the disease remains a significant public health problem. India has the highest burden of malaria in the South-East Asia Region, where *Plasmodium vivax* and *Plasmodium falciparum* are its main causes. While the two major malaria parasite species co-occur in India, their proportion varies across the country. Antibodies in an individual indicate current or past *Plasmodium* infection, and can be used to identify suitable vaccine candidates, as well as develop novel tools for malaria surveillance. We present the results of a pilot study undertaking the first large-scale characterization of antibody responses to ~1000 *Plasmodium* antigens at three field sites in India using high-throughput protein microarray technology. Individuals from the eco-epidemiologically diverse sites showed reactivity to 265 *P*. *vivax* and 373 *P*. *falciparum* antigens, regardless of infection status. Further comparison of individuals with symptomatic and asymptomatic malaria revealed the most immunogenic *Plasmodium* antigens, as well as antigens that were recognized with greater intensity in individuals that were asymptomatic at the point of sample collection. These results are a valuable addition to existing data from other malaria endemic regions, and will help to expand our understanding of host immunity against the disease.

## Introduction

The burden of malaria in India has halved over the last 15 years, yet India continues to account for over 70% of malaria cases in South East Asia [1]. The ‘National Framework for Malaria Elimination in India 2016–2030’ has two aims: eliminating malaria throughout the country by 2030 and maintaining malaria–free status in areas where transmission has been interrupted [2]. Long-lasting insecticide-treated bed nets and artemisinin combination therapy have greatly helped to reduce malaria incidence in India. However, as transmission declines, the proportion of asymptomatic and submicroscopic infections tends to rise in a population [3]; these infections can contribute to malaria transmission [4], but they remain undetected by the standard diagnostic and surveillance tools. In order to eliminate malaria, it will be critical to develop accurate and sensitive methods for diagnosis and surveillance of asymptomatic and submicroscopic malaria infections.

The human immune response to the malaria parasite *Plasmodium* is multi-faceted, involving both the humoral and cell-mediated response pathways. CD8+ effector T cells can kill intra-hepatocytic stages [5], while merozoites and intraerythrocytic stages are primarily controlled by antibody-mediated responses such as interference with invasion of naïve erythrocytes, increased clearance of antibody-bound erythrocytes, and antibody-dependent cellular cytotoxicity mechanisms [6, 7]. The importance of antibody-based responses against *Plasmodium* was first demonstrated by passive transfer of antibodies from a clinically immune adult to a symptomatic child, which conferred protection from severe disease [8, 9]. Antibodies are generated rapidly to several parasite antigens immediately following infection, boosted upon subsequent infections, and are able to persist for several years after parasite clearance [10, 11]. Despite being exposed to multiple infections, individuals living in endemic areas do not acquire sterile immunity to malaria; instead, they develop non-sterile transmission- and age-dependent protection from clinical disease, known as ‘naturally acquired immunity’ (NAI). Several studies have highlighted the role of antibody-based response in NAI, encompassing protection from infection (anti-parasite immunity) and severe clinical symptoms (anti-disease immunity). The acquisition of natural immunity has been extensively demonstrated for *P*. *falciparum*, and more recently for *P*. *vivax*, in regions of both high and low transmission [12, 13]. Interestingly, NAI is acquired more rapidly to *P*. *vivax* infection than to *P*. *falciparum* [12], which, hypothetically, could be attributed to the differing biology of the two parasite species, such as the ability of *P*. *vivax* to maintain a dormant state within hepatocytes [10]. Additionally, there may be a differential contribution of antibody responses to natural immunity against *P*. *vivax* and *P*. *falciparum* [14–19].

Antibodies in an individual can be indicative of recent exposure to *Plasmodium* parasites, current infections, or past infections, and can therefore be used to identify suitable candidates for vaccine development, and to develop tools that can estimate malaria transmission levels [20] or monitor the efficacy of treatment programs [21]. Antibody-based responses can be measured using techniques that assess responses to one or very few antigens, *e*.*g*., immunofluorescent antibody test, enzyme-linked immunosorbent assay *etc*., or that detect antibodies to several antigens simultaneously, such as genome-scale protein microarrays [20]. Previous attempts to characterize the antibody response to *Plasmodium* among Indian populations have been sparse [22], with only a few studies describing the targeted response to well-established vaccine candidates [23–26]. Protein microarrays have been used to characterize antibody responses in populations from malaria endemic regions in Africa, South East Asia, and South America [27]; however, there have been no such studies in India to date. To capture the diverse eco-epidemiology in India, we probed protein microarrays comprising ~500 *P*. *vivax* and ~500 *P*. *falciparum* antigens, with sera from individuals at three sites in India–Raurkela in the eastern state of Odisha, Nadiad in the north-west state of Gujarat, and Chennai in the southeastern state of Tamil Nadu. Although the two major malaria parasite species co-occur at all three sites, they differ in their prevalence. Historically, *P*. *vivax* has been predominant in Chennai and Nadiad, whereas *P*. *falciparum* has been predominant in Raurkela. The objectives of our study include: 1) to provide a descriptive analysis of seroreactivity profiles of individuals against *Plasmodium* antigens at three epidemiologically diverse malaria endemic sites in India; 2) to examine the relationship between age and the acquisition of antibodies against *Plasmodium* antigens; 3) to identify the most immunogenic *Plasmodium* antigens based on antibody responses at three sites in India; and 4) to identify antigens recognized with greater intensity by individuals with asymptomatic malaria.

## Materials and Methods

### Ethical statement

Ethical approval to conduct this study was obtained from New York University Institutional Review Board (Study #i10-00173) and the Ethics Committee of the National Institute of Malaria Research, India. All project staff completed Protection of Human Research Subjects training prior to beginning the study, and clinical samples were collected after informed consent was obtained from all participants. For all adult patients, informed written consent was obtained from literate patients, and oral consent (documented by a thumb print) was obtained from illiterate patients. For child participants, assent was obtained from the participant, in addition to written or oral consent from their parent or legal guardian.

### Study sites and sample collection

Samples were collected as part of epidemiology studies at three sentinel sites in India: [1] Chennai City in the state of Tamil Nadu; [2] Nadiad town and surrounding villages in the state of Gujarat; and [3] Raurkela town and surrounding villages in Odisha, during January 2013-April 2015. These field sites are part of a National Institutes of Health-funded International Centers of Excellence for Malaria Research (ICEMR).

Chennai is the largest city in the southern state of Tamil Nadu, with a population of over 7 million in 2011. Located on the eastern coast of India, it has a tropical wet and dry climate, with a rainy season focused between mid-October to mid-December. Chennai accounts for 55.6% of all malaria cases in Tamil Nadu, and had an annual parasite index (API) of 1.79 in 2013 [28]. Entomological inoculation rate (EIR) values are not available. *P*. *vivax* is the dominant species, and although transmission is perennial, malaria cases peak between July and October. Subjects were enrolled at the Besant Nagar Malaria Clinic, or in cross-sectional surveys conducted in the Besant Nagar catchment area, which is composed of middle- and upper- class urban dwellings, a few slums, and a large coastal fishing community.

Nadiad, with a population of 0.2 million in 2011 is located in the Kheda district in Gujarat state. Rainfall is primarily received between June and September. Nadiad is hypo-endemic for both *P*. *vivax* and *P*. *falciparum* species, with slightly higher prevalence of *P*. *vivax*, and an average API of 2.5 (range 0.87–4.12) and EIR of 0.05–0.21 [29]. The National Institute of Malaria Research (NIMR) Malaria Clinic enrolled subjects attending Nadiad Civil Hospital. In addition, subjects were enrolled in cross-sectional surveys conducted in rural areas in the vicinity of Nadiad town.

Raurkela, with a population of over 0.5 million, is located in Sundargarh district close to the northern border of the state of Odisha, and has a tropical climate, with high temperatures and heavy rainfall between June-September and December-January. *P*. *falciparum* is the dominant species in Raurkela, and it has the highest EIR (7.3–127) and API (average = 20, range 5.1–43.5) of the three field sites in our study [29]. Subjects were enrolled at a clinic set up in a suburb of Raurkela, and in cross-sectional surveys conducted in rural areas in the vicinity of Raurkela.

Sample collection and processing at these sites has been described in detail elsewhere [29–31]. To summarize, individuals between 1–69 years were enrolled after informed consent, and whole blood was collected to generate a blood smear for malaria diagnosis and to measure hemoglobin levels. The remaining blood was separated into plasma and red blood cell (RBC) fractions: DNA was extracted from the RBC fractions for species-specific detection of *Plasmodium* [32], and plasma samples were stored at -80°C.

### Pilot study design

Plasma samples from 353 individuals that were randomly selected from clinic and cross sectional studies at our field sites were utilized for this study (**Table 1)**. A total of 236 individuals were diagnosed as malaria-positive and 117 individuals were diagnosed as malaria-negative by microscopy and PCR. Individuals ≤ 15 years were categorized as children, and those > 15 years were categorized as adults. Of the 236 malaria-positive individuals diagnosed by PCR, 147 individuals with documented fever (body temperature ≥ 37.5°C) or a history of fever in the past 48 hours were categorized as symptomatic; 89 individuals without documented fever or history of fever in the past 48 hours were categorized as asymptomatic. Pooled plasma samples from 20 healthy semi-immune adults from Sepik, Papua New Guinea, collected in 2004 and where transmission of *P*. *falciparum* and *P*. *vivax* was equally high, were used as positive controls, and pooled plasma samples from unexposed individuals in the United States were used as negative controls.

**Table 1:** Summary of 353 samples collected at three sites in India.

	Raurkela	Nadiad	Chennai	Total
**All samples**	168	114	71	353
• Female	66	38	24	128
• Male	102	76	47	225
• Age (years)	2–69	6–69	15–60	**-**
• Species prevalence	*Pf > Pv*	*Pf < Pv*	*Pf < Pv*	**-**
**Malaria-negative**	48	44	25	117
**Malaria-positive**	120	70	46	236
***P*. *vivax (Pv)***	27	49	35	111
• Symptomatic	9	38	32	79
• Asymptomatic	18	11	3	32
***P*. *falciparum (Pf)***	86	19	11	116
• Symptomatic	40	17	7	64
• Asymptomatic	46	2	4	52
**Mixed infection**	7	2	0	9
• Symptomatic	3	1	0	4
• Asymptomatic	4	1	0	5

### Protein microarrays and probing

The Pf/Pv500 protein array (Antigen Discovery Inc., Irvine, CA) comprises a total of 515 *P*. *vivax* and 500 *P*. *falciparum* antigens, expressed in cell-free *in vitro* transcription/translation (IVTT) reactions. Accession numbers and description of the antigens are based on the *P*. *vivax* Salvador I and *P*. *falciparum* 3D7 genome annotation from the PlasmoDB database [33]. Complete array information is publicly available through the NCBI Gene Expression Omnibus database with accession number GPL18316. Sample processing and microarray probing was performed as described elsewhere [27].

### Data and statistical analysis

Microarray spot intensities were quantified using the ScanArray Express software (Perkin Elmer, Waltham, MA). The non-specific background signal for each spot was calculated as the median intensity of sample-specific no-DNA IVTT control spots. For data normalization, the raw intensity values for IVTT proteins were divided by the median intensity of corresponding IVTT controls and log_2_ transformed to generate median-normalized fold-over-control (FOC) values. To generate the heat map, median-subtracted intensity values were obtained by subtracting the median intensity of IVTT controls from the raw intensity of IVTT protein spots. Significance Analysis for Microarrays [34] was carried out by comparing the median-normalized intensity of antibody binding to Indian samples with unexposed controls from the United States, to determine antigens that are specifically recognized in the Indian cohort. Individual plasma samples were considered seropositive for a particular antigen if the corresponding log_2_(FOC) ≥ 1. The breadth of antibody response was determined as the number of antigens an individual or group of plasma samples were seropositive to, based on the above criteria. Comparison of the breadth of responses across various groups was carried out using the Kruskal Wallis test with p-values adjusted using Dunn’s correction for multiple testing. Analysis of differential antibody reactivity between groups was performed by a two-sample *T* test assuming unequal variance and corrected for false discovery rates using the Benjamini-Hochberg method. Statistical analyses were carried out using Microsoft Excel, R v3.0.1, and Prism v7.0. All data have been made publicly available through PlasmoDB [33].

## Results

### Evaluation of seroreactivity to *P*. *falciparum* and *P*. *vivax* antigens in India

We used the Pf/Pv500 protein microarray containing 515 *P*. *vivax* and 500 *P*. *falciparum* antigens to evaluate the seroreactivity of 236 malaria-positive and 117 malaria-negative individuals (353 total) from three study sites, Raurkela (n = 168), Nadiad (n = 114) and Chennai (n = 71), in India. **Table 1** provides characteristics of the individuals and study sites.

**Fig 1:**
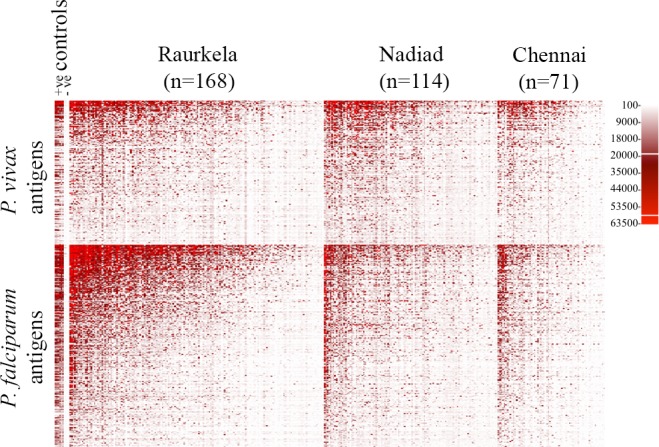
Serological profiles of malaria-positive and malaria-negative individuals from three sites in India. Heat map showing signal intensity of antibody binding to 265 *P*. *vivax* and 373 *P*. *falciparum* polypeptides in 236 malaria-positive and 117 malaria-negative samples collected from Raurkela, Nadiad and Chennai. Red indicates positive reactivity, white indicates no reactivity, and the gradient indicates intermediate reactivity. Samples for each site and *Plasmodium* antigens were ranked from left to right and top to bottom, respectively, by decreasing log_2_(FOC).

Comparison of antibody responses between all plasma samples identified 638 antigens recognized with significantly higher intensity in Indian samples versus unexposed U.S. controls. All subsequent analyses were carried out using this subset of 265 *P*. *vivax* and 373 *P*. *falciparum* antigens. The global profile of antibody binding to *Plasmodium* antigens at the three sites is shown **(Fig 1)**. The overall seropositivity or breadth of response against *P*. *falciparum* antigens was higher compared to *P*. *vivax* antigens.

### Variation in seroreactivity at three eco-epidemiologically diverse study sites in India

**Fig 2:**
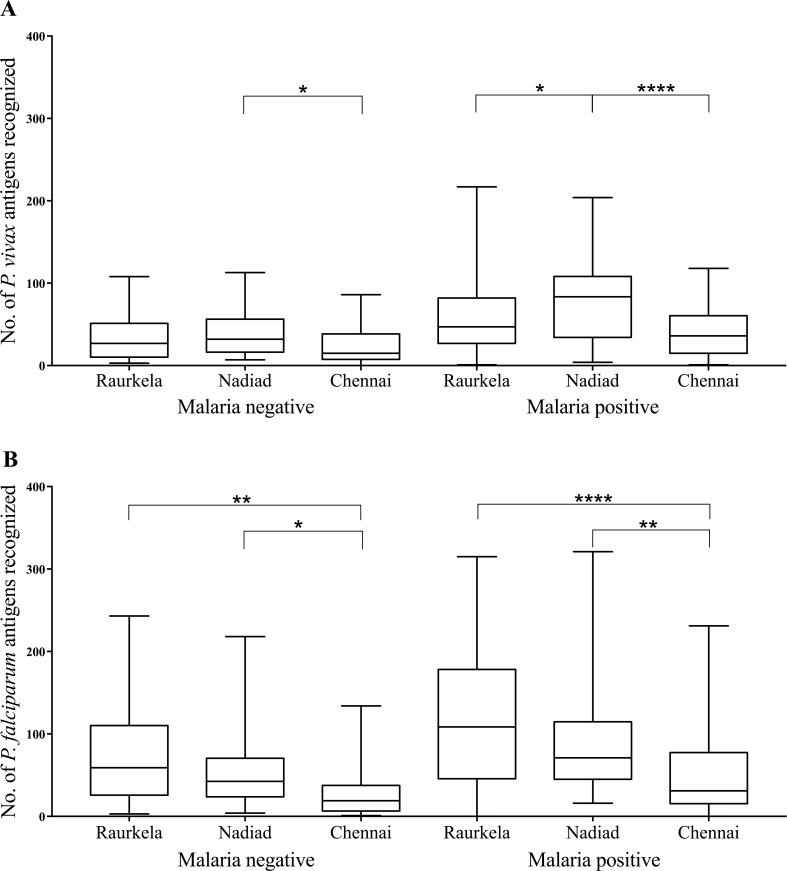
Breadth of antibody response to *P*. *vivax* and *P*. *falciparum*. Breadth of response to A) 265 *P*. *vivax* and B) 373 *P*. *falciparum* antigens in samples collected from malaria-positive (Chennai = 45; Nadiad = 55; Raurkela = 74) and malaria-negative adults (Chennai = 25; Nadiad = 34; Raurkela = 32) at three sites in India. The box indicates the first and third quartiles, the line inside the box indicates the median, and whiskers represent the minimum and maximum values. Kruskal–Wallis/Dunn adjusted p-values for pairwise comparison of groups are shown as asterisks: 0.03 (*), 0.002 (**), 0.0002 (***), <0.0001 (****).

To analyze observed differences in seroreactivity across the three Indian sites in more detail, we compared the breadth of response to *P*. *vivax* and *P*. *falciparum* antigens in adults. Malaria-positive adults from Nadiad showed significantly higher breadth of response to *P*. *vivax* antigens compared to malaria-positive adults from Raurkela (p < 0.03) and Chennai (p < 0.0001). Malaria-negative adults from Nadiad also showed higher levels (p < 0.03) of seropositivity to *P*. *vivax* than adults from Chennai **(Fig 2A)**. In the case of *P*. *falciparum* antigens, malaria-positive adults from Chennai had significantly lower seroreactivity compared to malaria-positive adults from Raurkela (p < 0.0001) and Nadiad (p < 0.002); a similar observation was made upon comparison between malaria-negative adults from Chennai with Raurkela (p < 0.002) and Nadiad (p < 0.03; **Fig 2B**).

### Age-dependent changes in antibody response to *Plasmodium* species

**Fig 3:**
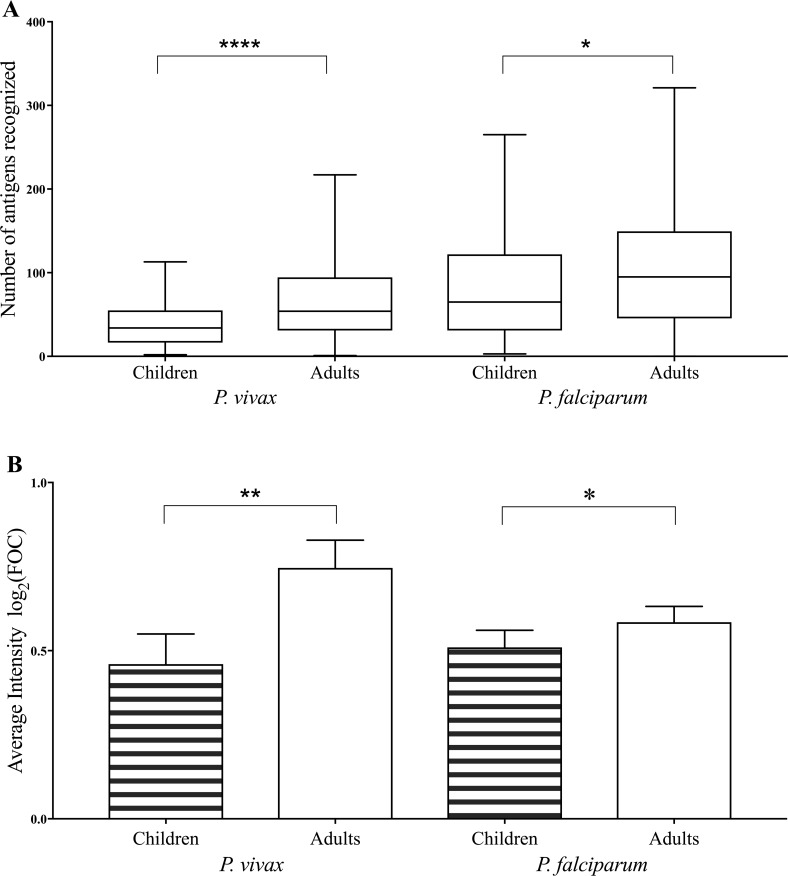
Age-dependent breadth and intensity of response to *P*. *vivax* and *P*. *falciparum*. Age-dependent A) breadth of response to 265 *P*. *vivax* and 373 *P*. *falciparum* antigens in children (n = 61) and adults (n = 129) from Raurkela and Nadiad. The box indicates the first and third quartiles, the line inside the box indicates the median, and whiskers represent the minimum and maximum values. B) Average of mean intensity of antibody binding to the same subset of *P*. *vivax* and *P*. *falciparum* antigens in children and adults, top of bars indicate the mean value and error bars represent 95% confidence interval of the mean. Kruskal–Wallis/Dunn adjusted p-values for pairwise comparison of groups are shown as asterisks: 0.03 (*), 0.002 (**), 0.0002 (***), <0.0001 (****).

We compared the breadth and intensity of antibody response between malaria-positive children (age ≤ 15 years) and adults (age > 15 years) to understand age-based differences in acquired immunity (limited to Raurkela and Nadiad due to insufficient numbers of children sampled in Chennai). Adults showed higher breadth of response against *P*. *vivax* (p < 0.0001; **Fig 3A**) and *P*. *falciparum* (p < 0.03; **Fig 3A**), as well as greater intensity of antibody binding against *P*. *vivax* (p < 0.002; **Fig 3B**) and *P*. *falciparum* (p < 0.03; **Fig 3A**) when compared to children.

### Highly immunogenic *Plasmodium* antigens in Indian samples

We identified highly immunogenic *Plasmodium* proteins across all study sites. For every antigen, we calculated the average reactivity and breadth of response in malaria-positive and malaria-negative samples at each site. We identified 26 *Plasmodium* antigens with average log_2_(FOC) ≥ 1 among malaria-positive samples at all sites; these antigens were also recognized by most of the malaria-negative samples at each site. This list of 14 *P*. *vivax* and 12 *P*. *falciparum* antigens represents the most immunogenic *Plasmodium* antigens that are indicative of current or past malaria infections **(Table 2)**. In the case of *P*. *vivax*, merozoite surface proteins, ETRAMP, SFT2, and a number of hypothetical proteins are recognized with the greatest intensity. Among the *P*. *falciparum* antigens recognized with the greatest intensity of antibody binding are members of the PfEMP1 family, PTP5, AMA1, and HSP70.

**Table 2:** Top immunogenic *Plasmodium* antigens.

Gene ID	Gene Description	ORF Fragment
***P*. *vivax***		
PVX_117680	hypothetical protein	Exon 1 of 2
PVX_092555	WD, Gbeta-repeat domain containing protein	Exon 1 of 6 Segment 1
PVX_116780	protein transport protein SFT2, putative	Exon 1 of 2
PVX_113825	transcription factor with AP2 domain(s), putative (ApiAP2)	Exon 1 of 1 Segment 1
PVX_083560	Plasmodium exported protein, unknown function	Exon 2 of 2
PVX_091935	hypothetical protein, conserved	Exon 2 of 3
PVX_097730	hypothetical protein	Exon 1 of 1
PVX_099980	major blood stage surface antigen Pv200	Exon 1 of 1 Segment 2
PVX_110935	hypothetical protein, conserved	Exon 1 of 1
PVX_090230	early transcribed membrane protein (ETRAMP)	Exon 1 of 2
PVX_114145	merozoite surface protein 10 (MSP10)	Exon 1 of 1
PVX_097625	merozoite surface protein 8, (MSP8)	Exon 1 of 1
PVX_084305	zinc finger protein, putative	Exon 1 of 1 Segment 1
PVX_118705	hypothetical, predicted Pf homolog liver stage antigen 3	Exon 1 of 1
***P*. *falciparum***		
PF3D7_1002100	EMP1-trafficking protein (PTP5)	Exon 2 of 2
PF3D7_0800200	erythrocyte membrane protein 1, PfEMP1 (VAR)	Exon 2 Segment 1
PF3D7_1007700	transcription factor with AP2 domain(s) (ApiAP2)	Exon 1 Segment 2
PF3D7_0422100	transmembrane emp24 domain containing protein	-
PF3D7_0420700	erythrocyte membrane protein 1, PfEMP1 (VAR)	Exon 2 Segment 1
PF3D7_0817300	asparagine-rich antigen	Exon 1 Segment 3
PF3D7_1133400	apical membrane antigen 1 (AMA1)	-
PF3D7_0315400	conserved protein, unknown function	Exon 1 of 1
PF3D7_0530100	SNARE protein, putative (SYN6)	Exon 2 of 2
PF3D7_0620400	merozoite surface protein 10 (MSP10)	Exon 1 of 1
PF3D7_0713900	conserved protein, unknown function	Exon 1 Segment 4
PF3D7_0818900	heat shock protein 70 (Hsp70)	-

### *Plasmodium* antigens recognized with greater intensity by adults with asymptomatic versus symptomatic malaria

**Fig 4:**
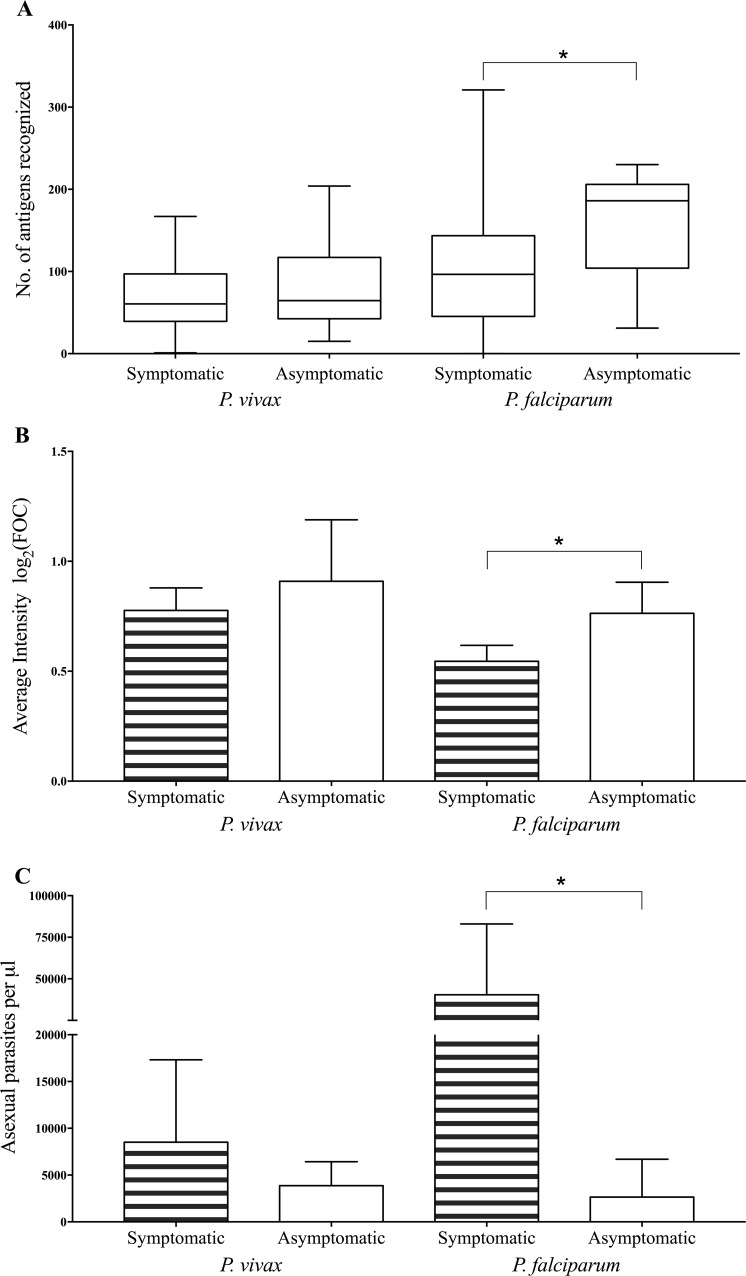
Comparison of individuals with symptomatic and asymptomatic malaria. A) Breadth of response to 265 *P*. *vivax* antigens, and 373 *P*. *falciparum* antigens in symptomatic (*P*. *vivax* = 58; *P*. *falciparum* = 38) and asymptomatic (*P*. *vivax* = 16; *P*. *falciparum* = 9) malaria-positive adults at three sites in India. The box indicates the first and third quartiles, the line side of the box indicates the median, and whiskers represent the minimum and maximum values. B) Average of mean intensity of antibody binding to 265 *P*. *vivax* and 373 *P*. *falciparum* antigens in symptomatic and asymptomatic malaria-positive adults, top of bars indicate the mean value and error bars represent 95% confidence interval of the mean. C) Average of *P*. *vivax* and *P*. *falciparum* asexual parasitemia (number of asexual parasites per microliter) in symptomatic and asymptomatic malaria-positive adults. Kruskal–Wallis/Dunn adjusted p-values for pairwise comparison of groups are shown as asterisks: 0.03 (*), 0.002 (**), 0.0002 (***), <0.0001 (****).

Based upon the presence or absence of fever at the time of enrollment and up to 48 hours earlier, *Plasmodium*-positive individuals were further categorized as symptomatic or asymptomatic. We compared the breadth and intensity of antibody response to *Plasmodium* antigens in adults classified into these two categories. Of the 174 malaria-positive samples, 121 were diagnosed by both PCR and microscopy, and 50 were detected only by PCR, *i*.*e*., were submicroscopic. Analysis of the 121 microscopy- and PCR-positive adults revealed that asymptomatic adults infected with *P*. *falciparum* showed significantly higher breadth (p < 0.03; **Fig 4A**) and intensity (p < 0.03; **Fig 4B**) of antibody response than symptomatic adults. In addition, we observed that the asexual parasitemia levels in asymptomatic adults were much lower (p < 0.002; **Fig 4C**) compared with symptomatic adults infected with *P*. *falciparum*. In the case of *P*. *vivax*, no significant differences in the breadth (**Fig 4A**) and intensity (**Fig 4B)** of antibody response were observed between adults with asymptomatic and symptomatic infection. The levels of asexual parasitemia were also comparable between asymptomatic and symptomatic adults infected with *P*. *vivax* (**Fig 4C**).

We were interested in identifying antigens that have significantly higher reactivity in sera from asymptomatic individuals compared to symptomatic individuals. We used log_2_(FOC) values to identify antigens that were differentially reactive between asymptomatic and symptomatic adults (p < 0.05, with Benjamini–Hochberg correction for false-discovery rate). Asymptomatic adults infected with *P*. *falciparum* displayed significantly higher reactivity to several *P*. *falciparum* antigens compared to symptomatic adults. We have identified 19 *P*. *falciparum* antigens that were recognized with 2-fold greater intensity in asymptomatic adults in India (**Table 3**). On the other hand, no *P*. *vivax* antigens showed significantly higher reactivity in asymptomatic compared to symptomatic individuals infected with *P*. *vivax*.

**Table 3:** *Plasmodium falciparum* antigens recognized with greater intensity in asymptomatic individuals.

Gene ID	Gene Description	Average log2(FOC)		Adjusted p-value
		Asymptomatic	Symptomatic	
PF3D7_0801000	exported protein (PHISTc), unknown function	2.77	1.39	4.49E-05
PF3D7_1036000	merozoite surface protein (MSP11)	2.81	1.44	3.71E-05
PF3D7_1335300	reticulocyte binding protein 2 homologue b (RH2b)	2.56	1.25	9.58E-05
PF3D7_1452000	rhoptry neck protein 2 (RON2)	2.60	1.29	9.87E-05
PF3D7_0207700	serine repeat antigen 4 (SERA4)	2.29	1.01	4.87E-04
PF3D7_0800300	erythrocyte membrane protein 1, PfEMP1 (VAR)	2.28	1.01	1.17E-04
PF3D7_0102200	ring infected erythrocyte surface antigen (RESA)	2.36	1.10	2.06E-04
PF3D7_0532100	early transcribed membrane protein 5 (ETRAMP5)	2.38	1.26	4.49E-05
PF3D7_0206800	merozoite surface protein 2 (MSP2)	2.35	1.24	2.06E-04
PF3D7_0702400	small exported membrane protein 1 (SEMP1)	2.43	1.32	1.53E-03
PF3D7_0831700	heat shock protein 70, putative (HSP70-x)	1.87	0.78	8.24E-04
PF3D7_0402400	exported protein, unknown function (GEXP18)	2.30	1.22	4.86E-04
PF3D7_0823300	histone acetyltransferase GCN5 (GCN5)	1.51	0.45	5.25E-03
PF3D7_1300300	erythrocyte membrane protein 1, PfEMP1 (VAR)	2.56	1.50	7.52E-04
PF3D7_1401400	early transcribed membrane protein 14.1 (ETRAMP14)	2.37	1.31	4.43E-04
PF3D7_1149200	ring infected erythrocyte surface antigen	2.35	1.29	7.70E-04
PF3D7_0220000	liver stage antigen 3 (LSA3)	3.00	1.95	1.58E-03
PF3D7_0933900	conserved protein, unknown function	2.46	1.43	1.82E-03
PF3D7_0207000	merozoite surface protein 4 (MSP4)	2.06	1.06	2.06E-03

## Discussion

We present the first genome-scale analysis of seroreactivity to *Plasmodium* antigens in the Indian population using protein microarray technology. The three field sites in our study are distributed across eco-epidemiologically diverse regions in India, with differing prevalence of *P*. *vivax* and *P*. *falciparum*. We observed a broad response to *P*. *vivax* and *P*. *falciparum* antigens at all three sites, regardless of malaria infection status. Overall levels of antibody binding were greater in *P*. *falciparum* antigens compared to *P*. *vivax* antigens, as previously reported in regions with co-endemic occurrence of *P*. *vivax* and *P*. *falciparum* [35]. Our Indian sample cohort showed significant seroreactivity to 265 *P*. *vivax* and 373 *P*. *falciparum* antigens compared to unexposed US controls.

In the case of *P*. *falciparum*, we observed that decreasing prevalence corresponded with a decrease in the breadth of antibody response. Malaria-negative adults showed a similar pattern to malaria-positive adults at all sites, reflecting the background seroreactivity due to parasite exposure. The breadth of response to *P*. *falciparum* antigens was significantly lower in adults from Chennai compared to both Raurkela and Nadiad; although the mean breadth of response appeared to be lower in Nadiad compared to Raurkela, the decrease was not statistically significant.

We did not observe a correlation between the breadth of antibody response and species prevalence for *P*. *vivax*. Malaria-positive adults from Nadiad showed significantly larger breadth of response than adults from both Chennai and Raurkela; this was not observed in malaria-negative adults. We were puzzled that regardless of their malaria infection status, the seroreactivity of individuals from Chennai was similar to Raurkela, which has much lower prevalence of *P*. *vivax*. Since we began the study in 2012, the incidence of malaria has gone down in India, particularly in the state of Tamil Nadu where Chennai accounts for ~55% of the malaria burden [28]. Thus, the reduced seroreactivity of Chennai individuals may be reflective of reduced transmission levels during the period of sample collection for our study. Interestingly, some Indian states, including Odisha and Gujarat, witnessed a spike in malaria cases in 2014 [36]. Therefore, the prevalence of *P*. *vivax* and *P*. *falciparum* in Raurkela and Nadiad may have been higher than expected during the course of our study. Hypothetically, *P*. *vivax* seroreactivity may also be affected by differential rates of *P*. *vivax* relapse; sites with a higher relapse rate may exhibit greater priming of the immune response. However, we do not have data on relapse rates at our three sites to address this conclusively. Additionally, the breadth of response could be affected by differences in the total number of individuals and the proportion of symptomatic and asymptomatic infections between the sites.

The breadth and magnitude of immune response have been known to increase with age, as a consequence of repeated exposure to the parasite. We compared the responses of children and adults from Nadiad and Raurkela to determine the influence of age on the immune response against malaria. The breadth and intensity of response against *P*. *vivax* as well as *P*. *falciparum* antigens was significantly higher in adults than in children.

Antigens recognized with the highest intensity by sera from both symptomatic and asymptomatic malaria patients may serve as indicators of exposure. Data from other ICEMR studies [27] have highlighted the need to identify country-specific indicators of exposure, as they may vary depending on the epidemiology of malaria in different parts of the world. As the prevalence of the two major *Plasmodium* parasite species varies across India, we were interested in identifying highly immunogenic antigens from all three sites that could be used to develop a serological assay for countrywide routine surveillance. It is also important to elucidate the kinetics of antibody acquisition and maintenance [37] in order to distinguish between recent versus past exposure. Markers of exposure in sera from children may be better indicators of recent exposure, as adults may have had several exposure events, confounding the evaluation of responses from recent exposure. Several of the top immunogenic *P*. *vivax* proteins in the Indian population, such as ETRAMP, MSP10, MSP8, and hypothetical proteins such as PVX_117680, PVX_083560, PVX_097730, PVX_110935, PVX_118705 have also been identified as highly immunogenic in other studies [38–42]. Among the top immunogenic proteins in *P*. *falciparum* identified in our study, members of the PfEMP1 family, PTP5, MSP10 and HSP70 were also identified as immunogenic or recognized with greater intensity in asymptomatic malaria in other ICEMR studies [13, 37, 43, 44]. Invasion-related proteins AMA1 and SYN6 were also highly immunogenic [45].

Asymptomatic individuals may not be protected from malaria parasite infection, but they may possess immunity against symptomatic disease. Our data indicate that adults with asymptomatic *P*. *falciparum* infection have lower average asexual parasitemia, but higher breadth as well as intensity of response, than adults with symptomatic infection. Asymptomatic *P*. *falciparum* infection was also associated with significantly higher seroreactivity to several *P*. *falciparum* antigens as compared to symptomatic infection, and these antigens may serve as novel vaccine candidates in addition to the limited repertoire of candidates currently being developed. A majority of the antigens associated with clinical immunity in *P*. *falciparum* infections are either exported to the infected red blood cell during the intraerythrocytic stages of parasite development (PfEMP1, RESA, ETRAMP, PHISTc, Hsp70-x, GEXP18) or present on the merozoite surface (MSP2, MSP4, MSP11, SERA4). These proteins are exposed to the host immune system for the longest duration of the infection, facilitating the development of a strong immune response. Some of these antigens play essential roles in vital processes such as invasion (RON2), making them promising vaccine candidates. GCN5 and LSA3, previously associated with protection from experimental challenge with sporozoites [46, 47], and invasion-related proteins, RON2 and RH2b, were recognized more strongly by asymptomatic individuals in our study MSP2, MSP4, MSP11, PHISTc, Rh2b, PfEMP1, LSA3 and SERA4 were also associated with protection from symptomatic disease in other malaria-endemic regions [13, 35, 43] demonstrating that the antigens associated with asymptomatic infection are common across populations, which is encouraging for the development of a vaccine.

Comparison of serological profiles of adults with symptomatic or asymptomatic malaria suggests that immunity to *P*. *falciparum* is associated with a broad and intense response to several antigens, but with low parasitemia levels. In contrast, adults with asymptomatic and symptomatic *P*. *vivax* infection had comparable breadth and intensity of antibody response, as well as similar asexual parasitemias. In addition, asymptomatic *P*. *vivax* infection was not associated with higher seroreactivity to specific *P*. *vivax* antigens. Based on these findings, it appears that unlike *P*. *falciparum*, antibody-mediated immune responses may have a much lower contribution in asymptomatic *P*. *vivax* infections. *P*. *vivax* is thought to be more pyrogenic than *P*. *falciparum*, since it stimulates a stronger fever-inducing cytokine response despite frequently presenting at a lower parasite burden than *P*. *falciparum* [48]. However, Goncalves *et*. *al*., propose that regulatory cytokines, such as IL-10 may play a more critical role in protecting *P*. *vivax* patients from severe clinical complications, while a strong inflammatory response may be critical in controlling parasite density in *P*. *falciparum* infections [14].

Our pilot study was conducted in tandem with other protein microarray projects within the ICEMR program and using the same Pf/Pv500 protein array [13, 27, 35, 43]. Together, these studies facilitate a global comparison of seroreactivity to over 1000 *Plasmodium* antigens, and provide a means to identify highly immunogenic proteins and antigens recognized more strongly in asymptomatic individuals, which may subsequently be used for the development of vaccines and routine surveillance tools. We acknowledge the limitations of using this array, since the antigens have been produced from sequence information of decades-old single reference isolates (*P*. *falciparum* 3D7 and *P*. *vivax* Salvador I). Our future studies will include country-specific redesign of the arrays using sequence data from diverse parasite strains circulating at our study sites, in order to capture regional diversity in antigenic genes. In addition, appropriate study design will be extremely important to tease out the complexities of the host immune response to malaria. In particular, prospective cohort studies, with careful monitoring of parasite transmission and patient serology, are likely to be the most informative. In conclusion, these data have broadened our understanding of naturally acquired immunity against *P*. *vivax* and *P*. *falciparum* in India, and will contribute to global malaria control and eradication measures.
